# Is whole‐body magnetic resonance imaging a source of anxiety in oncological patients?

**DOI:** 10.1002/cnr2.1737

**Published:** 2022-12-09

**Authors:** Ketti Mazzocco, Derna Busacchio, Paul Eugene Summers, Chiara Marzorati, Paola Pricolo, Giuseppe Petralia, Gabriella Pravettoni

**Affiliations:** ^1^ Applied Research Division for Cognitive and Psychological Science IEO European Institute of Oncology IRCCS Milan Italy; ^2^ Department of Oncology and Hemato‐Oncology University of Milan Milan Italy; ^3^ Division of Radiology IEO European Institute of Oncology IRCCS Milan Italy; ^4^ Precision Imaging and Research Unit ‐ Department of Medical Imaging and Radiation Sciences IEO European Institute of Oncology IRCCS Milan Italy

**Keywords:** anxiety, cancer patients, magnetic resonance imaging, personalized medicine, preferences, whole‐body MRI

## Abstract

**Objective:**

Magnetic resonance often produces feelings of anxiety before, or during, the examination. The aim of this study was to assess anxiety and potential causes of anxiety in cancer patients undergoing whole‐body magnetic resonance imaging (WB‐MRI).

**Methods:**

This monocentric study recruited 70 cancer patients who were scheduled to undergo WB‐MRI for detection, staging or therapy monitoring. At baseline (prior to the WB‐MRI), assessments were performed using the State–Trait Anxiety Inventory (STAI‐Y 1), Illness Perception Questionnaire (IPQ‐R), Big Five Inventory (BIF‐10) and Revised Life Orientation Test (LOT‐R), while at the end of the WB‐MRI examination the patients repeated the STAI‐Y 1 questionnaire and were asked to indicate their preference between WB‐MRI and computed tomography.

**Results:**

We found a positive correlation between pre‐ and post‐examination STAI‐Y 1 scores (*r* = 0.536, *p* < .0001), with no significant difference between them. Pre‐examination STAI‐Y 1 scores had a negative correlation with the emotional stability in the BIF‐10 questionnaire (*r* = −0.47, *p* = .001) and a positive correlation with emotional representation (*r* = 0.57, *p* = .001) in IPQ‐R. The post‐examination STAI‐Y 1 had a negative correlation with optimistic orientation (*r* = −0.59, *p* = .001).

**Conclusions:**

The anxiety associated with a WB‐MRI examination was only in small part associated with the examination itself, and in fact, most patients preferred WB‐MRI to computed tomography. Concern with the outcome of the examination was likely a greater source of anxiety.

## 1 BACKGROUND

Whole body magnetic resonance imaging (WB‐MRI) is an imaging method used for early disease identification and the monitoring of targeted therapy of several cancers.^1^ The implementation of WB‐MRI in oncology is currently recommended in international guidelines for the assessment of different cancer histotypes^2^, ^3^, ^4^ (including multiple myeloma,^5^ advanced stage melanoma,^6^ metastatic prostate cancer^7^) and cancer‐prone syndromes (Li‐Fraumeni,^8^ hereditary paraganglioma and pheocromocytoma syndromes^9^). Moreover, this exam is also widely used for the staging and follow‐up of other cancer histotypes and cancer related syndromes (including breast cancer,^10^ lymphoma,^11^ neurofibromatosis^12^ and Von Hippel Lindau Syndrome^13^). It is also commonly applied when standardized investigations have yielded inconclusive results,^14^ and in pregnant cancer women in need of accurate systemic staging.^15^

WB‐MRI allows the detection of small lesions throughout the body^16^ without the need for exposure to ionizing radiation and in most cases without the injection of contrast agents, thus avoiding their associated risks.^17^, ^18^ Moreover, WB‐MRI has a diagnostic performance equal to total‐body computed tomography (CT) with contrast agent or positron emission tomography (PET).^19^, ^20^ The combination of comparable performance and the non‐presence of risks from radiation exposure and contrast agent injection posed the WB‐MRI as a good alternative by radiologists for whole‐body examination of eligible patients.^21^ The literature shows however, that patients report discomfort and anxiety during magnetic resonance examinations and these factors can lead to the procedure being a stressful experience. A review investigating the relationship between anxiety symptoms and magnetic resonance imaging^22^ demonstrated that up to 30% of patients reported high levels of worry, while 5%–10% of patients showed severe psychological problems. The situation may be even more severe, as, Oliveri et al.^23^ found that 93.6% of patients who had previously undergone an MRI examination reported at least a medium level (*M* = 3.8 on a VAS scale) of anxiety and concern related to the examination.

In oncological patients, anxiety and concern arise in a context already charged with the fear of death and uncertainty: the need to control the disease and its prognosis often arise among patients despite cancer characteristics.^24^ Moreover, patients focusing on the negative consequences of the disease showed greater levels of anxiety. Conversely, Downe‐Wamboldt et al.^25^ have provided evidence that a favorable illness perception can allow a better comprehension of the disease, thus favor more effective coping strategies in cancer patients.

To the best of our knowledge, few qualitative studies investigating the experiences of patients undergoing an WB‐MRI examination have been conducted and only two studies have qualitatively explored patients' and subjects experience, acceptance and anxiety with WB‐MRI.^23^, ^26^, ^27^

The aim of this study was to assess anxiety with the State–Trait Anxiety Inventory (STAI‐Y 1)^28^ questionnaire and investigate potential factors affecting pre‐ and post‐exam anxiety in cancer patients undergoing whole‐body magnetic resonance imaging (WB‐MRI).

## 2 METHODS

This was a prospective study that examined the associations of breast and prostate cancer patients' disease perception, personality dimensions, and optimistic orientation with anxiety in the context of WB‐MRI.

### 2.1 Participants and procedure

Seventy oncological patients (46 Breast cancer; 24 Prostate cancer) undergoing WB‐MRI for staging and therapy monitoring were prospectively enrolled to this institutional review board approved study (1032_UID_1810) between June 2020 and November 2020. Written informed consent was obtained from all subjects involved in the study.

Participants included in the study were cancer patients with metastatic (stage IV) disease who were undergoing WB‐MRI based on an oncologist's recommendation with no contraindication to the MRI examination (e.g., pacemaker, pregnancy in the first trimester). Exclusion criteria were anxiety disorder and psychological or pharmacological treatments for anxiety.

Participants were recruited before the WB‐MRI, while they were waiting for the examination. After providing informed consent, the participants were invited to meet the psychologist for psychological assessment and collection of sociodemographic and medical characteristics, including gender and age.

The following measures were included in the psychological assessment:

- The State–Trait Anxiety Inventory subscale (STAI‐Y 1),^28^ which was the only questionnaire administered both before and after the WB‐MRI examination. It is a self‐report questionnaire composed of 20‐items having scores ranging from 1 to 4, with higher score indicating higher state anxiety levels.
- The Revised Life Orientation Test (LOT‐R)^29^ was administered before the WB‐MRI examination to measure the optimistic orientation. It is a self‐report questionnaire of 10 items that measures the optimism or pessimism of people's attitude toward the future. Scores for each item range from 0 to 4, with a higher total score in the sum of the items indicating more optimism.
- The 10‐item Big Five Inventory (BIF‐10)^30^ was used before the WB‐MRI examination to assess personality traits (agreeableness, conscientiousness, emotional stability, extroversion, and openness). It is a self‐report tool composed of 10 items, with scores ranging from 1 to 5.
- The Illness Perception Questionnaire (IPQ‐R)^31^ was also administered before the WB‐MRI examination to assess the patients' perception of illness. The questionnaire is composed by three sections: the identity subscale (assensing symptoms the patient associates with the illness), the causal subscale (measuring personal ideas about etiology), and a third section including different subscales on acute/chronic and cyclical timeline (the perceived duration of the illness), consequences (the expected effects and outcome), treatment control (how one controls or recovers from the illness), disease coherence, and emotional representations. It is a self‐report questionnaire with scores ranging from “strongly disagree” to “strongly agree”.

To evaluate the preferences about imaging technique, after the WB‐MRI examination the patients were asked to indicate which examination they preferred between WB‐MRI or CT. More specifically, patients answered the following question:” “If you had to repeat these tests in the future, would you choose CT or WB‐MRI?”. We note that the contexts of CT scans for the patients included both whole‐body examinations (with or without PET scan) for staging purposes, and local scans for treatment planning. In the former case, an injection is required either a PET radiotracer, or CT contrast agent depending on the specific examination undertaken. Whereas CT only scanners have a bore length much shorter than that of an MRI scanner (circa 50 cm vs 1.7 m), the CT‐PET scanner is comparable (1.5 m). The duration of CT‐only examinations (whole‐body or local) are typically less than 5 minutes whilst a CT PET examination lasts about 20 minutes, as compared to the roughly 35 minutes for WB‐MRI.

### 2.2 Statistical procedures

We calculated descriptive statistics of all the variables under analysis before performing a bivariate correlation analysis between the variables under study. All the analyses were performed with SPSS 26 (IBM Corp. Released 2019. IBM SPSS Statistics for Windows, Version 26.0.). A *t* test for dependent samples was performed to test for difference between pre‐ and post‐examination anxiety. Furthermore, a regression analysis was performed to test the impact of stable psychological variables such as personality characteristics (dispositional optimism and personality traits) and the illness perception on pre‐ and post‐examination state anxiety. Statistical significance was assigned at the two‐tailed 5% level.

## 3 RESULTS

### 3.1 Demographic characteristics of study sample

The 70 participants had a mean age of 60 years (range 37–82 years), 66% of patients were female with breast cancer, and 34% were male with prostate cancer. As regards education, 56% had graduate, 31% high school, and 13% middle school levels of education (Table 1).

**TABLE 1:** Patient characteristics

Characteristics	*N*	Mean	%/Range
Age	70	60.29	37–82
Disease group			
Breast	46		66%
Prostate	24		34%
Education level			
Middle school	9		13%
High school	22		31%
University	39		56%

### 3.2 Correlation and regression analysis

A positive correlation was found between pre‐ and post‐examination STAI‐Y 1 scores (*r* = 0.536, *p* < .001). There was however, no significant difference between the pre‐ and post‐examination STAI‐Y 1 scores (Table 2).

**TABLE 2:** Mean and correlation between pre‐ and post‐anxiety and personality characteristics, illness perception, and dispositional optimism

Correlation					
Questionnaire	Variable	Mean	SD	Anxiety pre‐MRI	Anxiety post‐MRI
				*r*	*r*
STAI‐Y 1	Anxiety pre‐MRI	42.49	18.01		0.54***
STAI‐Y 1	Anxiety post‐MRI	48.04	32.38	0.54***	
LOT‐R	Dispositional optimism	15.31	4.97	−0.33*	−0.59***
BIF‐10	Agreableness	7.22	1.69	−0.04	0.06
	Conscientiousness	8.07	1.71	−0.08	−0.05
	Emotional_stability	6.58	2.21	−0.47***	−0.32*
	Extraversion	6.11	1.43	0.01	0.06
	Openness	7.76	1.73	−0.14	0.11
IPQ‐R	Timeline	15.27	5.14	0.01*	0.08
	Consequences	14.31	4.79	0.35*	0.23
	Personal control	14.09	4.37	0.07	−0.18
	Treatment control	14.80	2.56	0.02	−0.14
	Disease coherence	14.33	3.72	−0.50**	−0.22
	Cyclical timeline	6.38	3.36	0.15	0.41**
	Emotional representation	12.38	6.35	0.57***	0.59***

*Note*: *p value ≤.05; **p value ≤.001; ***p value ≤.0001.

Negative correlations were found between pre‐examination STAI‐Y 1 and the emotional stability dimension in the BIF‐10 questionnaire (*r* = −0.47, *p* = .001), dispositional optimism in the LOT‐R questionnaire (*r* = −0.33, *p* = .05) and the disease coherence subscale score (*r* = −0.50, *p* = .001) in the IPQ‐R questionnaire.

The pre‐examination STAI‐Y 1 was positively correlated with the emotional representation subscale score (*r* = 0.57, *p* = .001), timeline (*r* = 0.01, *p* = .05), and consequences (*r* = 0.35, *p* = .05) in the illness representation section of the IPQ‐R.

Negative correlations were found between the post‐examination STAI‐Y 1 and dispositional optimism (*r* = −0.59, *p* = .001) of the LOT‐R questionnaire, and the emotional stability subscale (*r* = −0.32, *p* = .05).

The post‐examination STAI‐Y 1 was positively correlated with the cyclical timeline (*r* = 0.41, *p* = .01) and emotional representation (*r* = 0.59, *p* = .001) subscale scores in the illness representation section of the IPQ‐R.

Educational status, age and type of disease were not significantly correlated with the levels of anxiety seen in the pre‐examination STAI‐Y 1.

The multiple regression model with personality traits, dispositional optimism and illness perception (identity subscales) and type of pathology as predictors and pre‐examination anxiety as dependent variable produced *R* ^2^ = 0.390, *F* = 14.723, *p* < .001. The only variables having significant regression weights were from the illness representation section IPQ‐R questionnaire, namely: the emotional representation subscale score (*β* = 0.417, *p* = .003) indicating that patients with higher scores on this scale were expected to have higher level of anxiety before the exam, and the disease coherence subscale score (*β* = −310, *p* = .024), indicating that patients with higher scores on this scale were expected to have lower level of anxiety before the exam.

Regarding the level of anxiety post‐examination, the multiple regression model with personality traits and dispositional optimism and type of pathology as predictors produced *R* ^2^ = 0.334, *F* = 14.065, *p* = .001. The only variable that had a significant regression weight was the dispositional optimism of the LOT‐R questionnaire (*β* = −0.600, *p* < .001) indicating that patients with higher scores on this scale were expected to have lower level of anxiety after the exam.

Age did not have a significant effect on personality traits, dispositional optimism or illness perception.

### 3.3 Preferences of imaging technique

Of the 70 participants, 55 preferred WB‐MRI; 5 preferred CT, and 10 did not indicate a preference.

## 4 DISCUSSION

With the patient‐centered approach being of growing importance to oncology care, there are many aspects to everyday practice that require consideration of the patient's perspective, preferences and interests. In the present study, we examined the associations of breast and prostate cancer patients' disease perception, personality dimensions, and optimistic orientation with their anxiety in the context of WB‐MRI.

We found a positive correlation between pre‐ and post‐ WB‐MRI examination levels of anxiety, but no significant difference between them. We attribute the lack of significant difference to the fact that the patients had not yet received the examination report, and therefore the context was not resolved, when compiling the post‐examination STAI‐Y 1. This view is motivated by the findings by Oliveri et al.^24^ that the main concern reported by patients before undergoing a WB‐MRI examination was not related to the examination itself, but to the outcome, that is: the possibility of discovering the presence of cancer. Similarly, Katz et al.^32^ have found that along with the fear of pain, the expectation of the test results contributes to examination anxiety. Moreover, it is consistent with the correlation we found between pre‐examination anxiety and the “timeline”, “consequences” and “emotional representation” subscales of the Illness Perception Questionnaire, as these indicate that the intrusive thoughts about possible severe life‐threatening consequences of their illness and the related emotions are associated with the specific emotional state of patients while undergoing an examination that may confirm said concerns. Moreover, the idea that illness would last a long time and the lack of a coherent and complete comprehension of the disease are associated with higher state anxiety. In line with this, the patients with greater concerns about outcomes, also showed higher state anxiety levels. In fact, as most patients indicated a preference for WB‐MRI over CT, our results point to the WB‐MRI itself not being a strong factor of anxiety in our cohort.

As regards factors influencing the levels of pre‐ and post‐WB‐MRI examination anxiety, we found a negative association between dispositional optimism and pre‐ and post‐anxiety. This suggests that patients with higher levels of anxiety were less likely to expect good outcomes. A further negative correlation was observed between the emotional stability dimension in the BIF‐10 questionnaire and the pre‐ and post‐examination STAI‐Y 1. A lower emotional stability score in BIF‐10, indicates difficulty in controlling one's emotions.^30^ This suggests that anxiety in face of the WB‐MRI examination is, in part, related to the emotional aspects of the patient's act of undergoing the WB‐MRI examination. It is widely recognized that the need to deal with sensations of claustrophobia, face a noisy, sometimes uncomfortable environment, and stay still for several tens of minutes are factors that lead some patients to experiencing feelings of concern, discomfort to MR examinations.^33^, ^34^, ^35^ Indeed, these are sometimes so severe that patients experience their first claustrophobia attack during an MRI examination, even without a previous condition,^32^ and this could influence patients' perceptions.^36^ Reducing the time waiting for the medical report of examination would help reduce the anxiety experienced by patients.

The pre‐ and post‐WB‐MRI examination anxiety scores were both positively correlated with emotional representation of the illness representation section of the IPQ‐R. As the patients were not yet informed of the findings of their examination when compiling the post‐WB‐MRI assessment, and given the relatively high correlation between the pre‐ and post WB‐MRI levels of anxiety, it is unsurprising that factors related to disease perception (emotional representation) had a similar, positive association with anxiety before and after the WB‐MRI examination. Consistent with Zhang and colleagues (2016)^37^ who showed that a more negative emotional representation can be expected to drive negative emotional states; we saw illness perceptions and stress to be associated with the patients' anxiety. Elsewhere, it has been seen that illness perceptions play a significant role in emotional distress experienced by people with low‐grade brain tumors,^38^ but did not play a significant role in positive affect.

Interestingly, the regression model showed none of the personality characteristics to predict patients' anxiety experienced before the examination. Indeed, the only significant predictors were factors associated with patients' illness perception. In particular, independently of the type of cancer, the patients who were the most concerned about their disease (measured by emotional representation subscale), and were less able to make a sense of the disease (measured by disease coherence subscale), had a higher probability to experience high level of anxiety.

The post‐examination anxiety showed associations with cyclical timeline and emotional representation subscales of the illness representation section of IPQ‐R and the dispositional optimism measured by means of LOT‐R. These aspects likely become relevant, or are unmasked, once the patient's act of undergoing the examination has been completed.

Notably, we found no associations between type of disease or level of education and level of anxiety. This is somewhat surprising given evidence in other situations that sociodemographic (educational status, age, working status) and clinical characteristics (stage of the disease, time of diagnosis) are important determinants of illness perceptions.^39^ Our results indicate a negative correlation of anxiety post‐examination with optimistic orientation; this is in line with previously published studies that showed high levels of pessimism are risk factors for anxiety and depression.^40^ Interestingly, dispositional optimism was the only variable we found that predicted the level of patients' anxiety after the examination. The more optimistic the patient, the lower the anxiety that he/she experienced. This last finding, together with the significant contribution of the emotional representation and the coherence patients attribute to their disease have relevant clinical implications. All these variables are modifiable factors. Clinical psychological and relaxation interventions can be implemented to improve optimism and modify the mental representation of the disease and associated emotions that in turn will affect the level of anxiety before and after WB‐MRI examination.^41^, ^42^

Several limitations of our study are worth mentioning. First, only two types of disease were present in the patients (breast and prostate cancer), and second, our cohort was relatively small and derived from a single clinical center. Taken together these considerations may limit the generalizability of our findings. Further, WB‐MRI is still not available in many centers, but with the growing evidence in favor of wider use of WB‐MRI for staging and therapy monitoring of cancer patients argues for its greater use, lending clinical relevance to our findings. Lastly, a STAI‐Y questionnaire was not performed after the patients had received the findings of the WB‐MRI examination. This would have provided stronger evidence for the role of examination outcome in determining post WB‐MRI anxiety.

## 5 CONCLUSIONS

The anxiety experienced by patients undergoing a WB‐MRI examination was only in small part associated with the examination itself, and in fact, most patients preferred WB‐MRI to CT. Concern for the consequences of their disease and, possibly, for the outcome of the examination was likely a greater source of anxiety. The mental representation of the disease in its emotional and cognitive (sense‐making) component have a crucial weight on anxiety, as well personality disposition such optimism. Interventions aimed at improving illness perceptions and reducing perceived stress may also be effective in improving the psychological health and quality of life of patients.

## AUTHOR CONTRIBUTIONS

**Ketti Mazzocco:** Conceptualization (lead); formal analysis (lead); methodology (lead); writing – original draft (equal); writing – review and editing (equal). **Derna Busacchio:** Conceptualization (equal); data curation (equal); investigation (lead); methodology (equal); writing – original draft (equal); writing – review and editing (equal). **Paul Eugene Summers:** Data curation (equal); writing – original draft (equal); writing – review and editing (equal). **Chiara Marzorati:** Data curation (equal); formal analysis (equal); writing – review and editing (equal). **Paola Pricolo:** Supervision (equal); writing – original draft (equal); writing – review and editing (equal). **Giuseppe Petralia:** Conceptualization (equal); funding acquisition (equal); methodology (equal); project administration (equal); supervision (equal). **Gabriella Pravettoni:** Funding acquisition (equal); project administration (equal); resources (equal); software (equal); supervision (lead).

## ETHICS STATEMENT

The present study was approved by the IEO Ethics Committee (Trial ID IEO1032).

## FUNDING INFORMATION

This study was funded by FIEO‐CCM. This work was partially supported by the Italian Ministry of Health with Ricerca Corrente and 5 × 1000 funds.

## CONFLICT OF INTEREST

The authors have stated explicitly that there are no conflicts of interest in connection with this article.

## Data Availability

The datasets used and analyzed during the current study are available from the corresponding author.
